# The influence of an emotion regulation intervention on challenges in emotion regulation and cognitive strategies in patients with depression

**DOI:** 10.1186/s40359-024-01949-6

**Published:** 2024-09-20

**Authors:** Mohamed Hussein Ramadan Atta, Mervat Mostafa El-Gueneidy, Ola Ahmed Rashad Lachine

**Affiliations:** Psychiatric and mental-health nursing Department, Faculty of Nursing, Alexandria University, 9 Edmond Vermont Street - Smouha, Alexandria City, Egypt

**Keywords:** Cognitive emotion regulation, Depression, Emotion regulation difficulties, Emotion regulation module

## Abstract

**Background:**

Patients with depression struggle with significant emotion regulation difficulties, which adversely affect their psychological well-being and hinder recovery. Traditional therapeutic approaches often fail to adequately address these challenges, leading to a persistent gap in effective mental health care. This research seeks to address this gap by investigating the impact of emotion regulation skills training on patients with depression.

**Aim:**

To assess the difficulties in emotion regulation among patients with depression and evaluate the impact of an emotion regulation skills training intervention on those with higher levels of emotion regulation difficulties, specifically focusing on increasing the use of adaptive emotion regulation strategies and reducing the use of maladaptive emotion regulation strategies.

**Method:**

A quasi-experimental research design was utilized, using three tools: a socio-demographic and Clinical Data structured interview schedule, Difficulties in Emotional Regulation Scale, and Cognitive Emotion Regulation Questionnaire. Eighty patients with depression were recruited to assess those with higher levels of emotion regulation difficulties; out of those with greater difficulties, 30 patients were chosen to participate in the emotion regulation skills training intervention.

**Result:**

The 80 studied subjects' emotion regulation difficulties scores ranged from 158 to 169 (164.5 ± 3.21), and they indicated less use of adaptive cognitive emotion regulation strategies and more use of maladaptive cognitive emotion regulation strategies (56.07 ± 2.67). Regarding the intervention group, the overall mean score of the 30 patients’ emotion regulation difficulties decreased from 167.35 ± 2.21 pre-intervention to 105.85 ± 3.33 post-intervention (*p* < 0.0001). Cognitive emotion regulation total scores improved markedly from 54.07 ± 1.66 to 35.2 ± 3.46 (*p* < 0.01).

**Implication:**

Healthcare providers should routinely assess emotion regulation difficulties in patients with depression and integrate personalized treatment plans that target individual emotion regulation difficulties.

**Conclusion:**

The findings suggest that the emotion regulation intervention has the potential to improve emotion regulation difficulties and cognitive emotion regulation strategies among patients with depression.

## Introduction

Major Depressive Disorder (MDD) is a mental health state marked by ongoing reactions of sadness, loss of hope, and diminished interest in activities that used to be pleasurable [1]. MDD is a prevalent disorder [2], where individuals experience challenges in effectively managing and controlling their emotions [3]. Moreover, it was reported that MDD impacts approximately 3.28% of adults worldwide, with the prevalence rising to 4% [4]. Globally, it is estimated that around 280 million people suffer from depression [5].

In addition, research has demonstrated a rise in depression during the COVID-19 outbreak; a combination of increased stress, social isolation, disrupted routines, and exposure to trauma can severely impair emotional regulation, leading to heightened vulnerability to depression during the pandemic [6]. A systematic review revealed that the prevalence of depression during the COVID-19 pandemic was 33.7% among a sample of 44,531 individuals [7].

Depression is now broadly acknowledged as a complex, multifactorial disorder manifesting through affective, cognitive, and psychosocial symptoms [8, 9]. The varied nature of MDD complicates the understanding of the interrelationships among these dimensions, emphasizing the necessity of a multimodal approach for effective management and treatment that considers the intricate interactions between affective, cognitive, and psychosocial domains [10].

Depression is associated with challenges in emotional processing, including difficulties in recognizing, accepting, comprehending, and managing emotions [11, 12]. These difficulties contribute to effectively responding to and regulating emotions, leading to a persistent and distressing emotional pattern characterized by elevated negative emotions, diminished positive emotions, and maladaptive emotion regulation (ER) [3, 13].

Individuals with depression often engage in maladaptive emotion regulation strategies (ERS), where efforts to manage negative experiences may backfire, leading to an increase in symptoms. ER involves modulating which emotions one experiences, the timing of these emotions, and how one feels or expresses them [14, 15]. Research indicates that those with MDD tend to use more maladaptive and fewer adaptive emotion regulation strategies [12]. Additionally, difficulties with emotion regulation appear to continue even after individuals have recovered from MDD [16, 17].

Regarding adaptive ERS, it was found that individuals currently experiencing depression tend to use less reappraisal and acceptance of emotions compared to those without depression [18]. This suggests that individuals with depression may have difficulties in emotional awareness, clarity, and tolerance. Emotional awareness and clarity challenges can make it harder for individuals to identify and regulate their emotions effectively [19]. Individuals with depression often report lower emotional awareness [20], less clarity of negative feelings [21], and higher levels of alexithymia compared to those without depression [22]. Additionally, limited emotional tolerance, characterized by being easily overwhelmed by emotions, may lead individuals to rely more on maladaptive emotion regulation strategies like avoidance or suppression to manage their emotions [23].

Concerning the maladaptive ERS, Individuals with MDD often use ineffective ERS and have difficulty implementing effective ones, which contributes to their struggles in down-regulating sad moods [24]. Rumination, as a maladaptive emotion regulation strategy in MDD, tends those individuals to dwell on negative thoughts and feelings, and this tendency is associated with depressive symptoms [25]. They tend to ruminate more than healthy individuals [9, 15], which can intensify and prolong feelings of depressed mood, increasing the risk of developing or relapsing into depressive episodes [26]. Despite its adverse effects, individuals with MDD, both currently and in remission, may perceive rumination as a helpful strategy [27].

In addition, suppression is where individuals attempt to suppress both positive and negative emotions. People with MDD tend to use suppression more than healthy controls, and habitual use of suppression is linked to depressive symptoms and increased rumination [25]. Moreover, avoidance is considered a maladaptive emotion regulation strategy associated with depression, where individuals avoid specific stimuli or situations to prevent emotional reactions. People with MDD report avoiding emotional experiences more than those without depression, and this avoidance is associated with depressive symptoms [28].

Given the role of dysfunctional ERS and less use of adaptive ones in the development of depressive symptoms [3, 24–28] and the moderate effectiveness of existing treatments [29–33], emotion regulation modules targeting maladaptive ERS may be particularly beneficial in the context of depression [33–35]. In the literature review by Saccaro et al. (2024), the three most frequently studied psychotherapeutic interventions for emotion regulation were Dialectical Behavior Therapy (DBT) in 48% of original articles, Cognitive Behavioral Therapy (CBT) in 38% of reviews, and Mindfulness-Based Cognitive Therapy (MBCT) in 33% of articles. The most commonly used clinical measures to assess emotion regulation outcomes were the Difficulties in Emotion Regulation Scale (DERS), utilized in 81% of original articles, and the Cognitive Emotion Regulation Questionnaire (CERQ), used in 20% of original articles. In our study on depression, we are focusing on the emotion regulation module of DBT and will use DERS and CERQ as critical variables to measure outcomes [36].

One such intervention is dialectical behavior therapy (DBT), including the emotion regulation module, which is effective in treating depression [37]. Hammouda et al. (2020) conducted a systematic review of DBT's effectiveness for depression; they found it to be a promising treatment option for depression [38, 39]. Despite the existing use of DBT for emotion regulation difficulties, there remains a significant gap in research explicitly focusing on the emotion regulation module of DBT and its unique impact on emotion regulation difficulties and cognitive emotion regulation strategies among patients with depression. Most studies have examined DBT as a whole rather than isolating its components. Consequently, there is a need to assess the unique contribution of the emotion regulation module.

The significance of this study lies in enhancing our understanding of the mechanisms underlying emotion regulation in depression, contributing valuable insights into the specific challenges faced by patients in managing their emotions. The study provides empirical evidence on the effectiveness of emotion regulation skills training in clinical practice for patients with depression. It guides the development of personalized treatment plans that address the unique needs of patients with depression. The potential to enhance overall mental health and well-being by equipping patients with effective emotion regulation strategies is a crucial outcome, as it may reduce the severity and frequency of depressive symptoms.

This study aims to investigate the emotion regulation profile of patients with depression and determine the impact of an emotion regulation module on emotion regulation difficulties and cognitive emotion regulation strategies. Specifically, it will assess the baseline levels of emotion regulation difficulties among patients diagnosed with depression and identify the common cognitive emotion regulation strategies employed by these patients. The study will also evaluate the effectiveness of a structured emotion regulation skills training module in reducing emotion regulation difficulties and improving the use of adaptive cognitive emotion regulation strategies.

The quasi-experimental includes a more extensive and diverse sample, which can enhance the generalizability of the findings to a broader population. A quasi-experimental design is instrumental in mental health research, where ethical considerations and logistical challenges often preclude random assignment.

### Objective

1. Assess the difficulties in emotion regulation among patients with depression
2. Evaluate the impact of an emotion regulation skills training intervention on patients with significant emotion regulation difficulties, specifically focusing on:
  Increasing the use of adaptive emotion regulation strategies.
  Reducing the use of maladaptive emotion regulation strategies.

### Research hypothesis

Patients with depression who receive emotion regulation skills training will demonstrate significantly lower levels of emotion regulation difficulties and will employ more adaptive emotion regulation strategies compared to patients with depression who do not receive such training.

## Methodology

### Research design

This research integrates both descriptive and quasi-experimental methods to provide a more complete understanding of emotion regulation difficulties and the impact of the intervention.

### Setting

The study was conducted in the Psychiatric Outpatient Clinic of El-Mery University Hospital in Alexandria, Egypt. This Hospital is a prominent healthcare institution affiliated with Alexandria University and operates under the Ministry of Higher Education, Egypt.

The Psychiatric Outpatient Clinic at El-Mery University Hospital offers free treatment services, making mental health care accessible to patients from diverse socioeconomic backgrounds. The clinic is staffed by a multidisciplinary team of healthcare professionals, including psychiatrists, psychologists, psychiatric nurses, and social workers, all dedicated to delivering high-quality mental health services. This clinic serves as a critical resource for the local community, addressing various neuropsychiatric conditions such as depression, anxiety, schizophrenia, and bipolar disorder.

The clinic's commitment to providing accessible services ensures that financial barriers do not impede patient participation, facilitating a more prominent and representative sample for the study.

El-Mery University Hospital serves a diverse patient population, many relying on the clinic's free treatment services. Adopting a quasi-experimental design allowed us to leverage existing resources and infrastructure efficiently. It also ensured that the study could proceed within the available budget and staffing constraints while minimizing any potential burden on patients already receiving care.

### Participant’s recruitment

The study was conducted at the Psychiatric Outpatient Clinic of El-Mery University Hospital, Alexandria, Egypt. Based on outpatient records, approximately 1 to 3 patients with depression visit the clinic daily, averaging 36–48 patients per month. Using the Epi Info 7 program to estimate the sample size, the study used the following parameters: a population size of 150 patients with depression over three months, 50% expected frequency, 10% acceptable error, and a 99% confidence coefficient. The sample size formula used was *n* = Z2⋅p⋅(1 − p)e2n = \frac{{Z^2 \cdot p \cdot (1—p)}}{{e^2}}*n* = e2Z2⋅p⋅(1 − p)​, where the Z-value (2.576 for 99% confidence), pp is the expected frequency (0.50), and he is the acceptable margin of error (0.10). Substituting these values, the initial sample size was calculated to be approximately 166. To adjust for the finite population size of 150, the finite population correction (FPC) formula nadj = n1 + n − 1Nn_{\text{adj}} = \frac{n}{1 + \frac{n—1}{N}}nadj​ = 1 + Nn − 1​n​ was applied, yielding an adjusted sample size of about 79.02, which was rounded up to 80. This sample size ensures high confidence and precision in the study's findings. The study ultimately included 80 patients with depression for the assessment of their emotion regulation profile, with 30 of these patients recruited to receive the emotion regulation skills training intervention.

Participants were selected using a convenient sampling method, following the criteria:

### Inclusion criteria

- Participants were diagnosed with depressive disorders based on the DSM-V, confirmed using The Structured Clinical Interview for DSM-V for both the control and study groups (1).
- Diagnosed with comorbid substance abuse disorder.
- Aged from 20 to less than 60 years

### Exclusion criteria

- Engaged in any form of psychotherapy at least 1 year before initial assessment.
- Participants' age exceeds 60 years.

**Fig. 1:**
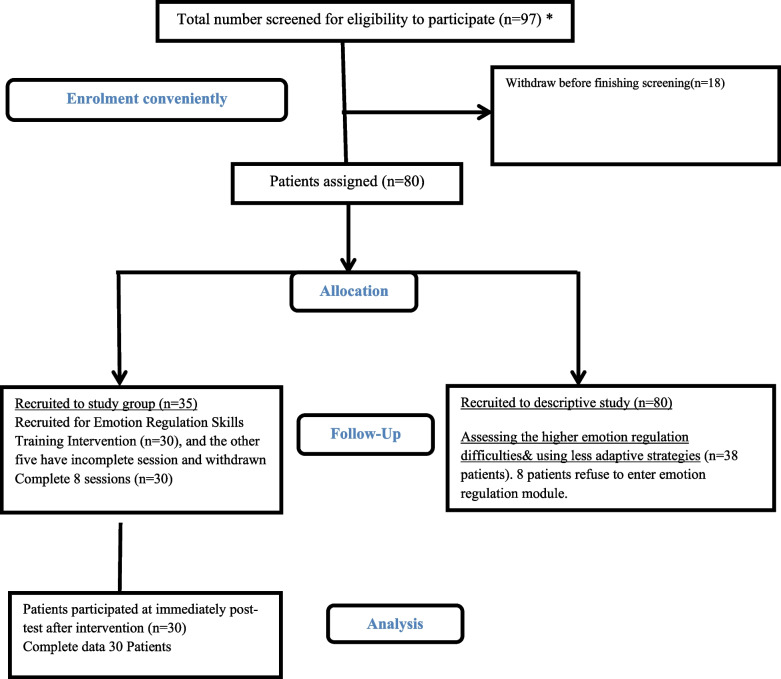
Flow diagram

Figure 1 illustrates the recruitment process. Initially, 97 participants were approached, with 80 patients successfully recruited to assess the emotion regulation profile. Of the 80 patients recruited to assess the patients with higher levels of emotion regulation difficulties (60 males and 20 females), 30 (22 males and eight females) were chosen to participate in the emotion regulation skills training intervention.

However, 18 patients withdrew before completing the screening, necessitating replacements to maintain the sample size of 80. Among these, 35 patients scored higher on the Difficulties in Emotion Regulation Scale (DERS) (scores above 108), indicating less adaptive and more maladaptive emotion regulation strategies. Ultimately, 30 patients participated in the eight-session Emotion Regulation Skills Training Intervention and received the usual routine care provided by the hospital. The remaining five patients were excluded due to interrupted follow-up and withdrawal from therapy.

### Ethical considerations

The study adhered to strict ethical guidelines to ensure the protection and well-being of all participants. The study protocol was reviewed and approved by the Ethics Committee of the Faculty of Nursing, Alexandria University (IRB00013630/109/9/2022). The approval ensured that the study design and procedures complied with ethical standards for research involving human participants. All participants were provided detailed information about the study's purpose, procedures, potential risks, and benefits. Written informed consent was obtained from each participant before their inclusion in the study. Participants were assured that their participation was voluntary and that they could withdraw from the study without any consequences to their treatment.

Participants' privacy and confidentiality were strictly maintained throughout the study. Personal identifiers were removed from all data, and results were reported in aggregate form to prevent the identification of individual participants.

The study was designed to ensure that participants would not be exposed to harm. The emotion regulation skills training intervention was intended to benefit participants by improving their emotion regulation strategies. Regular monitoring and support were provided to address any issues or concerns during the intervention. All data collected during the study were securely stored and accessible only to the research team. Data protection measures were in place to prevent unauthorized access and ensure data integrity.

By following these ethical guidelines, the study aimed to uphold the highest ethical research standards, ensure all participants' safety, rights, and well-being, and contribute valuable insights into the effectiveness of emotion regulation skills training for patients with depression.

### Measures

**Tool I** The demographic and clinical information was chosen based on its relevance to understanding the patients' socio-economic background and clinical history, which are critical for comprehensive psychiatric evaluation and treatment planning. Selecting specific socio-demographic variables, such as age, biological sex, marital status, educational level, employment status, and residence, provides a holistic view of the patient's life context, which can influence their mental health and treatment outcomes. For instance, age and sex can affect the prevalence and presentation of psychiatric disorders. At the same time, marital status, educational level, and employment status can impact the patient's social support systems and economic stability, which are essential for recovery. The place of residence can also be crucial, as it may reflect access to healthcare facilities and community resources.

The clinical data, included the patient's diagnosis, duration of illness, age of onset of illness, and number of previous psychiatric hospitalizations. This information helps understand the chronicity and management history of the patient's illness, which is essential for tailoring individualized treatment plans. For example, the duration of illness and age of onset can provide insights into the disease trajectory. At the same time, the number of hospitalizations can indicate the illness's severity and the effectiveness of past interventions.

**Tool II** is the Difficulties in Emotional Regulation Scale (DERS), a self-report scale composed of 36 items developed by Gratz and Roemer (2004). The scale items are categorized into six general subscales: non-acceptance of emotional responses, difficulty engaging in goal-directed behaviors, impulse control difficulties, lack of emotional awareness, limited access to emotional regulation strategies, and lack of emotional clarity. The DERS provides a total score ranging from 36 to 180, with higher scores indicating more significant emotional difficulties. A total score equal to or above 108 reveals more significant emotional regulation difficulties. The scale has good internal consistency, with α > 0.80 for all subscales [40].

**Tool III** is the Cognitive Emotion Regulation Questionnaire (CERQ), a 36-item questionnaire developed by Garnefski et al. (2002) that assesses cognitive emotion regulation strategies individuals use after experiencing adverse life events or situations. The questionnaire consists of nine subscales grouped into adaptive strategies (five subscales) and less adaptive strategies (four subscales). The adaptive strategies are acceptance, positive refocusing, planning, positive reappraisal, and putting into perspective, while the less adaptive strategies are self-blame, rumination, catastrophizing, and blaming others [41]. Each subscale consists of four items with a five-point Likert scale ranging from 1 (rarely) to 5 (almost always). The total score of CERQ is classified into three groups: low (36 to 83), moderate (84 to 131), and high (132 to 180). The less adaptive strategy items were reversed to obtain the total score of CERQ. The total adaptive subscale scores range from 20 to 100 and are divided into three groups: low (20 to 46), moderate (47 to 73), and high (74 to 100). The total less adaptive subscale score ranges from 16 to 80 and is divided into three groups: low (16 to 36), moderate (37 to 58), and high (59 to 80). The CERQ is internally consistent with Cronbach's alpha coefficients, which are, in most cases, over 0.80 and have good factorial validity, discriminative properties, and construct validity [41].

The reliability and validity of the DERS and CERQ were rigorously evaluated to ensure their robustness as data collection tools in the study. The original authors could translate the DERS and CERQ into Arabic, followed by back translation to ensure linguistic accuracy and consistency. Face validity was confirmed through expert review by professionals in psychiatric nursing, psychology, and psychiatry, ensuring that the scale effectively measures the intended constructs in the context of depression.

Additionally, the DERS and CERQ underwent reliability assessment on ten patients with depression through internal consistency measures, revealing Cronbach's alpha coefficients of 0.87 and 0.84 across all subscales, indicating strong reliability. A pilot study conducted on ten patients with depression validated the questionnaire's construct validity and applicability. Subjects involved in the pilot study and internal consistency test were excluded from the main study to prevent data duplication.

### Study procedure

The preparation phase of this study involved several meticulous steps to ensure the robustness and cultural appropriateness of the research instruments and intervention. Before the study, researchers completed a comprehensive 22-h online training program on Dialectical Behavior Therapy (DBT) and Linehan's Emotion Regulation Module, supervised by Dr. Ahmed M. Abdel Karim, the Linehan Institute ambassador for Egypt and the Middle East [42].

Recognizing the importance of cultural adaptation, the Emotion Regulation Module training manual was reviewed and adapted for the Egyptian context, including translation into Arabic and revision of session content and exercises to align with local cultural norms. The research instruments underwent rigorous validation, including the newly developed Socio-Demographic and Clinical Data Structured Interview Schedule and translated instruments (DERS and CERQ).

The veracity of the demographic and clinical information was verified through a combination of patient interviews and the review of medical records. Patients were asked to provide accurate and detailed responses to each question during the structured interview. To ensure reliability, the information obtained from the patients was cross-checked with their medical records, which included documented diagnoses, treatment histories, and hospitalization records. This triangulation method helps to confirm the accuracy of the data and minimizes the risk of self-report bias or inaccuracies in patient recall. Additionally, any discrepancies between patient reports and medical records were addressed through follow-up questions and clarifications during the interview process, further ensuring the validity of the collected data.

The principal author met patients to collect data in paperwork sheets. After eliciting Tool 1, Patients with higher emotion regulation difficulties and less adaptive strategies were kept on the list after manually calculating Tools II and III. The list contained 35 patients; the researcher explained the study's goal and emotion regulation skills. Whenever the number of recruited patients reached 5–7, the researchers implemented emotion regulation skills in that group. Detailed explanations about the study and intervention were provided to each participant before obtaining informed consent.

Data collection began on January 5, 2023, and concluded on May 18, 2023. It encompassed baseline assessments, intervention delivery (over eight sessions for the study group), and post-test assessments using DERS and CERQ administered individually to intervention groups to evaluate outcomes Table 1.

**Table 1:** Application of the emotion regulation skills intervention were

**Session**	**Specific objectives**	**Content and processes**
Part I: Name and understanding emotions. **Session 1: **week 1&2	- Develop the ability to describe emotions. - Learning to observe, describe, and name emotions can help regulate emotions. - Detect myths about emotions and practice challenges against them.	The patient demonstrated skills and homework assignments about: **I-** **Pros and Cons of Changing Emotions**: Learning to observe, describe, and name your emotions can help the patient to regulate emotions. The patient made a list of the pros and cons of changing the emotion you are having difficulty with and another list of the pros and cons of not changing your emotion. **II-** **Emotion diary**: The patient wrote and recorded the strongest, longest-lasting, and painful emotions as an emotion (either the strongest emotion of the day, the longest-lasting one, or the one that was the most painful or gave the most trouble). **III-** **Myths about emotions:** For each myth, write down a challenge that makes sense to you. Although the one already written may make much sense, try to come up with another one or rewrite the one there in your own words.
**Part II**: **Change emotional responses.** **Session 2: **week 3&4	1- Develop the ability to oppose negative emotions and consequences. 2- Follow the steps of problem-solving.	✤ **Check the facts** Determine whether the event is causing emotion, interpretation of the event, or both. Use mindfulness skills of observing and describing. Observe the facts, and then describe the facts observed. ✤ **Opposite Action to Change emotions** (Opposite body language and opposite words): Figure out what would be opposite actions, and then do the opposite actions. Remember to practice the opposite action all the way. For example, **anger** Do the OPPOSITE of your angry action urges. For example: 1. GENTLY AVOID the person you are angry with (rather than attacking). 2. TAKE A TIME OUT and breathe in and out deeply and slowly. 3. BE KIND (rather than mean or insulting). ✤ **Problem-solving to Change emotions:** Select a prompting event that triggers a painful emotion. Select an event that can be changed. Turn the event into a problem to be solved.
**Part III**: **Reducing Vulnerability to Emotion Mind** **Session 3: **week 5&6	1- Develop the ability to rethink the negative. 2- Develop a list of positive experience 3- State values that energized action	1- The** ABC **model described**: ** ■ **A as A**ccumulate positive emotions: Make changes in life so that positive events will happen more often in the future. e.g., Working on my car, Planning a career ■ **B as B**uild mastery: Do things that make you feel competent and effective to combat helplessness and hopelessness. ■ **C as C**ope ahead: Rehearse a timetable so you are prepared to cope skillfully with emotional situations. 2- **The PLEASE** skills model elaborated as □ **PL** as **a** treatment for physical illness □ **E** as balance **E**ating □ **A** as avoid mood-altering substances □ **S** as balance **S**leep □ **E** as get **E**xercise 3- Patient demonstrated Skills and homework assignments about: ➢ **Pleasant events Diary** The patient was instructed that accumulating pleasant events can take planning. For each day of the week, write down at least one possible pleasant activity or event. ** ➢ Getting from Values to Specific Action Steps** The patient made a list of several of the most important values. ➢ **Diary of Daily Actions on Values and Priorities** for tracking progress in reaching goals and living according to one's values. You can either fill out one page for each value or goal you are working on or fill it out every day, no matter what goal you are working on. ➢ **Build Mastery and Cope Ahead** The patient was instructed to write plans for practicing mastery and write what they did to increase their sense of mastery. ➢ **Putting ABC Skills together Day by Day** Write down what you plan to do that day at night or first thing in the morning; as you go or at the end of the day, write down what you did. ➢ **Practicing PlEASE Skills** The patient was instructed to write down what they did to practice the PLEASE skills. ➢ **Target Nightmare Experience Forms** o Changed Dream Experience Form o Dream Rehearsal and Relaxation Record o Sleep hygiene Practice Sheet
**Part IV**: **Reducing Vulnerability to Emotion Mind** **Session 4: **week 7&8	- Develop the ability to cope with the negative emotions. - Develop the ability to observe emotions. - Develop a plan for how to deal with the inability to do skill	**Mindfulness of Current Emotions** The patient learned to ➢ observe his/her emotions. ➢ practice mindfulness of body Sensations ➢ Do not necessarily act on your emotions. **Troubleshooting Emotion Regulation Skills** When you cannot get your skills to work, try doing so to see if you can figure out what is going wrong.

### Statistical analysis

The data collected in this study utilized IBM SPSS software version 20.0. Various statistical tests were applied based on the nature of the variables and the study design. The statistical analysis of this research utilized various methods to analyze the data collected from the subjects. Descriptive statistics, including frequencies, percentages, means, and standard deviations, were employed to summarize the socio-demographic and clinical characteristics of the participants. To ensure the appropriateness of our statistical methods, we applied the Shapiro–Wilk test to check the normality distribution of our sample. Upon applying the Shapiro–Wilk test, our results indicated that the data followed a normal distribution. Therefore, we proceeded paired t-test to compare the means of the same group, of normally distributed quantitative variables. at two different times—specifically before and after the emotion regulation skill intervention. η^2^ = Partial Eta Squared is used to measure Effect Size: It indicates the magnitude of the effect of an independent variable on the dependent variable, which is essential for understanding the practical significance of results beyond just statistical significance. This test was applied to the Difficulties in Emotional Regulation Scale (DERS) and the Cognitive Emotion Regulation Questionnaire (CERQ) scores to determine if there were statistically significant changes. The results showed significant reductions in all DERS subscales and the overall DERS score post-intervention, with highly significant *p*-values (*p* < 0.01, *p* < 0.001). Similarly, significant improvements were observed in all CERQ subscales and the overall CERQ score post-intervention, with highly significant *p*-values (*p* < 0.01, *p* < 0.001). Standard deviations were reported alongside mean scores to understand data dispersion.

## Results

### The demographic and clinical information

Table 2 presents the distribution of the total subjects according to their socio-demographic characteristics. It appears that the total number of subjects in this study was 80 patients with depression. The percentage of male patients constituted 75% of the subjects. The age of the studied subjects ranged between 21 and 55 years. Patients aged 20 to 30 years constituted 48.8% of the total subjects. Regarding marital status, married subjects amounted to 41.3% of the subjects, and single subjects reached 37.5%. The subjects were either divorced (17.5%) or widowed (3.75%).

**Table 2:** Distribution of the total subjects according to their socio-demographic characteristics

Patients' socio-demographic Characteristics	Total subjects: 80	
	**No**	**%**
**Sex**		
Male	60	75.0
Female	20	25.0
**Age (years)**		
20–30	39	48.8
31–40	21	26.3
41- less than 60	20	25.0
Min. – Max	21.0 – 55.0	
Mean ± SD	34.61 ± 8.40	
**Marital status**		
Single	30	37.5
Married	33	41.3
Divorced	14	17.5
Widow	3	3.75
**Education status**		
Illiterate	7	8.75
Read and write	14	17.5
Primary education	14	17.5
Secondary	35	43.75
University	10	12.5
**Occupation**		
Un-employed	40	50.0
Employed	25	31.2
Housewife	15	18.8
**Place of Residence**		
Rural	10	12.5
Urban	70	77.5
**Birth order**		
Only child	11	13.75
First child	19	23.75
Middle child	38	47.5
last-child	12	15.0
**Family size**		
1 – 3	40	50.0
4 – 6	30	37.5
7–	10	12.5
**Income**		
Not enough	30	37.5
Enough	50	62.5
**Cohabitation**		
Alone	17	21.2
With family	63	78.8

*SD *Standard Deviation

Table 3 presents the distribution of the total subjects according to their clinical characteristics. The duration of illness among studied subjects ranged between 4 months and 25 years. 37.5% of the total subjects had a duration of illness from 4 months to one year. For 25% of the subjects, the duration of illness ranged between 10 and 25 years. The majority of subjects (91.2%) had no previous hospitalization.

**Table 3:** Distribution of the total subjects according to their clinical characteristics

Patient's clinical characteristics	Total subjects: 80	
	**No**	**%**
**Duration of illness (in years)**		
4 m-1 year 1- 2 years 2–10	30 12 18	37.5 15.0 22.5
10–25	20	25.0
**Min. – Max. Mean ± SD**	**4 M – 25.0 12.30 ± 4.76**	
**Previous psychiatric hospitalization**		
** Yes**	**7**	**8.75**
No	73	91.2
**Family History**		
** Yes**	**52**	**65.0**
No	28	35.0
** If Yes**	No:52	
***Family Diagnosis***		
Depression	37	71.2
Bipolar Disorder	15	28.8
***Family/patient consanguinity***	No:52	
1st	31	59.6
2nd	21	40.4
**Family Support**		
** Yes**	**65**	**81.3**
No	15	18.8
** If Yes**	No:65	
**Type of Support**		
Financial	17	26.2
Psychological	17	26.2
Financial and Psychological	31	47.6

*SD *Standard Deviation

### Emotion regulation among patients with depression

Table 4 shows the distribution of the total subjects according to their Difficulties in Emotional Regulation score (DERS). It is worth mentioning here that higher total and subscales scores indicate higher difficulties in emotional regulation. The table denotes that the studied subjects’ overall scores ranged from 158 to 169, with a mean score of 164.5 ± 3.21.

**Table 4:** Distribution of the total subjects according to their Difficulties in Emotional Regulation scores (total and subscales scores)

**DERS total and subscales scores**		**Min. – Max**	**Mean ± SD**	**Total subjects: 80**			
				**Scoring Low**^a^		**Scoring high**^b^	
Subscales	Range						
				**No**	**%**	**No**	**%**
**Non-acceptance of emotional responses**	(6–30)	29.0 – 30.0	29.80 ± 0.40	16	20.0	64	80.0
**Difficulty engaging in goal-directed behaviors**	(5–25)	24.0 – 25.0	24.74 ± 0.44	21	26.3	59	73.8
**Impulse control** **difficulties**	(6–30)	21.0 – 30.0	27.75 ± 3.09	17	21.3	63	78.8
**Lack of emotional** **awareness**	(6–30)	10.0 – 24.0	18.65 ± 5.40	30	37.5	50	62.5
**Limited access to emotional regulation strategies**	(8–40)	38.0 – 40.0	39.55 ± 0.53	35	43.8	45	56.3
**Lack of emotional clarity**	(5–25)	21.0 – 25.0	24.03 ± 1.52	15	18.8	65	81.3
***Overall, DERS***	***(36–180)***	***158.0 – 169.0***	***164.5*** ** ± ** ***3.21***	***0***	***0***	***80***	***100***

*DERS *Difficulties in Emotional Regulation Scale, *SD *Standard Deviation

^a^Scoring less than mid-point

^b^Scoring at mid-point or higher

Regarding DERS subscales, the highest percentages of subjects’ emotional regulation difficulties were in “*Lack of emotional clarity*” (81.3%) and “*Non-acceptance of emotional responses*” (80%). These were followed by “*Impulse control difficulties*” (78.8) and “*Difficulty engaging in goal-directed behaviors”* (73.8%).

Table 5 displays the distribution of subjects according to their Cognitive Emotion Regulation results (totaland subscales scores).

**Table 5:**
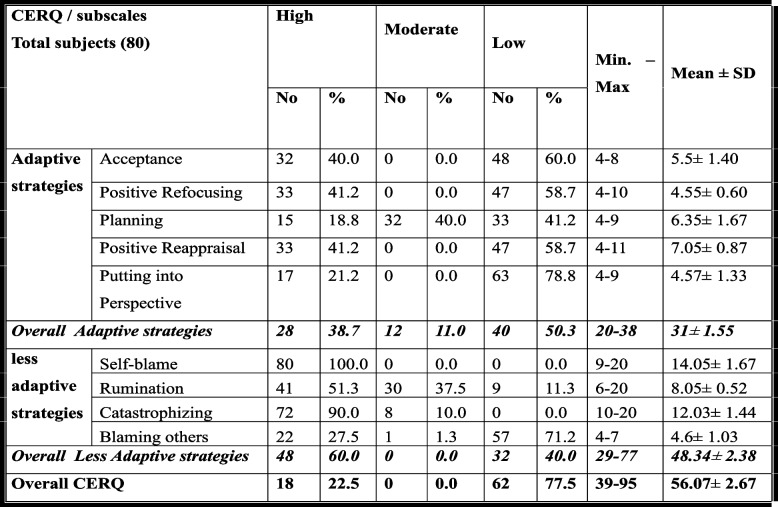
Distribution of the total subjects according to their Cognitive Emotion Regulation scores (total and subscales scores)

*SD* Standard Deviation

The subjects' overall scores indicated less use of adaptive cognitive emotion regulation strategies and more use of maladaptive cognitive emotion regulation strategies. The total overall scores ranged from 39 to 95, with a mean equal to 56.07 ± 2.67.

Subjects' scores regarding adaptive strategies ranged between 20 and 38, with a mean of 31 ± 1.55. For those with a higher percentage of adaptive cognitive emotion regulation, putting into perspective was the highest (78.8%), followed by Acceptance (60%).

Concerning the less adaptive strategies, the subjects' overall scores indicated less use of adaptive cognitive emotion regulation, ranging from 29 to 77 and a mean of 48.34 ± 2.38. Those who scored highest were Self-blame and Catastrophizing (100% and 90%, respectively).

### Effect of emotion regulation Module on emotion regulation difficulties

In supporting our hypothesis, patients with depression who receive emotion regulation skills training will demonstrate significantly lower levels of emotion regulation difficulties compared to patients with depression who do not receive such training. Table 6 displays the mean difference of study subjects before and after conducting emotion regulation skills intervention. It can be noticed that the mean score of the overall subjects decreased from *167.35* ± *2.21* in pre-intervention to *105.85* ± *3.33* post-intervention*,* with a high statistically significant difference *(p < 0.0001).*.

**Table 6:** The mean difference of studied subjects according to Difficulties in Emotional Regulation scores pre and post-emotion regulation skill intervention

**DERS (study subjects: 30)**	**Pre-intervention** **Mean ± SD**	**Post-intervention** **Mean ± SD**	**Paired t-test of significance**	P/η^2^
Non-acceptance of emotional responses	29.85 ± 0.40	17.74 ± 0.44	13.623 ***(p*** ** < ** 0.0001** ***)***	< 0.0001** /0.258
Difficulty engaging in goal-directed behaviors	24.74 ± 0.44	12.74 ± 2.50	14.03 (p < 0.001*)	< 0.001** 0.292
Impulse control difficulties	28.75 ± 3.40	10.95 ± 2.40	15.703 ***(p*** ** < ** 0.0001** ***)***	0.001** /0.133
Lack of emotional awareness	21.35 ± 5.44	17.35 ± 3.24	8.601 (p < 0.01*)	< 0.01* /0.18
Limited access to emotional regulation strategies	39.55 ± 0.53	20.25 ± 2.36	18.082 (p < 0.001*)	< 0.001** 0.272
Lack of emotional clarity	24.25 ± 1.52	16.22 ± 3.52	12.623 (p < 0.1)	0.1 /0.101
Overall, DERS	167.35 ± 2.21	105.85 ± 3.33	24.188 ***(p*** ** < ** 0.0001** ***)***	< 0.0001** /0.258

p: *p*-value for comparing between pre-test and post-test

Paired t-test of significance: dependent t-test η^2^= *Partial Eta Squared (Small Effect: Around 0.01, Moderate Effect: Around 0.06, Large Effect: Around 0.14)*

*SD *Standard deviation

^**^Statistically significant at *p* ≤ 0.001

^*^Statistically significant at *p* ≤ 0.01

The mean score of non-acceptance of emotional responses decreased from *29.85* ± *0.40*. to *17.74* ± *0.44* after the intervention. Also, impulse control difficulties declined from *28.75* ± *3.40* pre-intervention to *10.95* ± *2.40* post-intervention, with a statistically significant difference in both subscales (***p*** < ***0.0001).*** Regarding the lack of emotional awareness subscale, the mean score decreased from 17.0 – 24.0 to 14.0 – 20.0 post-intervention with a statistically significant difference (***p*** < ***0.01).***

Table 7 matches our hypothesis that patients with depression who receive emotion regulation skills training will employ more adaptive emotion regulation strategies compared to patients with depression who do not receive such training. This table compares the before and after demonstrations of emotion regulation skills according to the mean difference between the studied subjects. Regarding the adaptive cognitive emotion regulation strategies, the mean score of Putting into Perspective increased from 4.57 ± 1.01 to 14.7 ± 2.41 post-intervention with high statistically significant (*p* < 0.0001). As for Positive Refocusing, it can be noticed that the mean score elevated from 4.55 ± 0.60 to 17.5 ± 1.3 after the program, with a statistically significant difference (*p* < 0.001).

**Table 7:** The mean difference between the studied subjects' cognitive emotion regulation pre- and post-emotion regulation skill intervention

**CERQ Subscale**(study subjects:30)	**Pre-intervention**	**Post-intervention**	**Paired t-test of significance**
Acceptance	*4.5* ± *1.28*	*5.5* ± *2.21*	5.023 ***(p*** **<** 0. 01*)
Positive Refocusing	*4.55* ± *0.60*	*17.5* ± *1.3*	10.71 ***(p*** **<** 0.001*)
Planning	*5.01* ± *1.60*	*10.22* ± *0.6*	3.65 ***(p*** **<** 0.01*)
Positive reappraisal	*4.35* ± *1.07*	*15.75* ± *2.3*	8.44 ***(p*** **<** 0.01*)
Putting into perspective	*4.57* ± *1.01*	*14.7* ± *2.41*	6.55 ***(p*** **<** 0.0001**)
Self-blame	*12.05* ± *1.52*	*7.25* ± *3.02*	2.623 ***(p*** **<** 0.01*)
Rumination	*13.05* ± *1.72*	*14.23* ± *0.9*	4.178 ***(p*** **<** 0.01*)
Catastrophizing	*18.77* ± *1.74*	*11.2* ± *3.04*	3.23 ***(p*** **=** 0.003*)
Blame others	*4.1* ± *0.63*	*4.1* ± *0.63*	2.01 ***(p*** **<** ***2.01)***

Paired t-test of significance: dependent t-test

*SD *Standard deviation

p: *p*-value for comparing between pre-test and post-test

^**^Statistically significant at *p* ≤ 0.001

^*^Statistically significant at *p* ≤ 0.01

Speaking of the less adaptive cognitive emotion regulation strategies, the Catastrophizing mean score decreased from 18.77 ± 1.74 to 11.2 ± 3.04 post-intervention with a statistically significant difference of *p* = 0.003. Also, Self-blame and Rumination decline from 12.05 ± 1.52 and 13.05 ± 1.72 before intervention to 7.25 ± 3.02 and 14.23 ± 0.9 after program skills.

Table 8 shows the mean difference between studied subjects’ overall cognitive emotion regulation and overall subscales scores about pre and post-emotion regulation skill intervention. The mean overall cognitive emotion regulation score decreased from *54.07* ± *1.66* pre-intervention to *35.2* ± *3.46* post-program with a statistically significant difference (*t *= 8.452*, p* < *0.01)*. Regarding the overall adaptive strategies mean score*,* it can be noticed that the pre-intervention score dropped from *25* ± *2.55 to 67* ± *3.05* after the program, with a statistically significant difference (*t* = 18.651*, p* < *0.001)*.

**Table 8:** The mean difference between the studied subjects' overall cognitive emotion regulation and overall subscales scores pre- and post-emotion regulation skill intervention

**CERQ (study subjects:30)**		**Pre-intervention**	**Post-intervention**	**Paired t-test of significance**	**P/η**^2^
**Overall Adaptive strategies**	**Min. Max**	20.0- 36.0	56.0 -83.0	18.651 ***(p*** ** < ** 0.001*)	< 0.0001** /0.262
	**Mean ± SD**	*25* ± *2.55*	*67* ± *3.05*		
**Overall, less Adaptive strategies**	**Min. Max**	30.0 – 77.0	26.0- 51	8.06 (p < 0.001*)	< 0.001** 0.272
	**Mean ± SD**	*51.66 – 2.08*	*30.41* ± *2.11*		
**Overall CERQ**	**Min. Max**	38.0 – 95.0	30.0–77.0	8.452 ***(p*** ** < ** 0.01*)	< 0.01* 0.195
	**Mean ± SD**	*54.07* ± *1.66*	*35.2* ± *3.46*		

Paired t-test of significance: dependent t-test

*SD *Standard deviation

p: *p*-value for comparing between pre-test and post-test

^**^Statistically significant at *p* ≤ 0.001

^*^Statistically significant at *p* ≤ 0.01

Also, the overall mean score of less adaptive strategies increased from *51.66 – 2.08 to 30.41* ± *2.11* post-intervention with a statistically significant difference (*t* = 8.06*, p* < *0.001)*.

## Discussion

Depression is characterized by emotion regulation difficulties that contribute to the onset and maintenance of depression [3]. Emotion regulation difficulties manifest in various forms, such as an inability to control impulsive behaviors when distressed, a lack of emotional clarity, and difficulty accepting negative emotions. These difficulties not only intensify the severity of depressive episodes but also hinder recovery and increase the risk of relapse [11–13]. This study aims to investigate the effect of the Emotion Regulation Module on emotion regulation difficulties and cognitive emotion regulation strategies among patients with depression. Specifically, it seeks to address the following questions.

### Emotion regulation among patients with depression

The results of this study revealed that most of the studied subjects with depression have high emotion regulation difficulties. These findings are consistent with many previous studies [15, 16, 18, 40, 41]. Emotion regulation difficulties among the studied patients could be attributed to impaired processing of positive and negative emotions. Emotion processes include attention to and perceiving information that could elicit emotional responses [43–45]. They also include subsequent emotional arousal to such stimuli and the expression and experience of emotions. Patients with depression tend to experience increased attention to negative affect, reduced perception of positive affect, and less cognitive reappraisal to modulate negative emotions [15]. These mood disturbances are accompanied by negative affective biases during the appraisal, refocusing, and interpretation of emotional information [46, 47]. The current study supports the link between emotion processing and cognitive processes. It revealed that patients use less adaptive cognitive emotion regulation strategies, such as putting into perspective, positive refocusing, and positive reappraisal. These strategies may be linked to difficulties in attention, perception, and appraisal of emotions.

Another reason for emotion dysregulation among studied subjects could be the interaction between working memory and emotions. It has been proposed that the experience of negative mood is generally associated with the activation of mood-congruent representations in working memory [44]. Thus, emotion regulation difficulties are related to more frequent negative thoughts, selective attention to harmful stimuli, and greater accessibility of negative memories, leading to rumination of these memories [48]. This explanation is also supported by the results of the current study, which showed a higher frequency of rumination among the studied subjects. Difficulties in controlling the access of mood-congruent material to working memory are associated with increased ruminative thinking and maladaptive emotion regulation [49].

The present study's findings also reveal that the studied subjects have difficulty with emotion regulation related to the acceptance of emotions. Different authors emphasized that the inability to accept or value emotional reactions and avoidance of distressful internal experiences may create several emotion dysregulations [50, 51]. In this respect, for emotions to be regulated effectively, they require understanding and acceptance of self-emotional responses [52]. The present study reveals a high prevalence of lack of self-awareness among the studied subjects.

### Effect of emotion regulation Module on emotion regulation difficulties

As regards the application of emotion regulation skills intervention for patients with depression, the findings of the intervention resulted in significant positive change in emotion regulation difficulties. These findings are consistent with previous studies worldwide [30, 53–57].

Menefee (2020) concluded that patients should be equipped with techniques to manage their emotional responses, likely improving their well-being and decreasing mental health symptoms directly [58]. Patients learn adaptive coping with emotions by enhancing their abilities to accept, tolerate, and modify painful emotions. They learn to name and understand their emotions and change emotional responses when possible [59]. In addition, using mindfulness skills during the intervention affected patients’ emotional responses. It helped them manage difficult ones (using a wise mind instead of an emotional mind [60, 61]. The current study also showed a higher frequency of adaptive emotion regulation strategies and less frequency of maladaptive ones after emotion regulation skills intervention.

Moreover, the intervention helped the study subjects improve their refocus on planning and putting things into perspective. These results could be attributed to the ability of cognitive control, which allows patients to direct their attention away from thoughts that might otherwise upset them and focus on managing difficult emotions. This ability has a significant impact on emotion regulation [62, 63]. The literature reveals that when cognitive control fails, patients with depression may find themselves lacking control impulses and scattered without planning [64, 65]. In this respect, emotion regulation skills intervention enhances cognitive control by teaching patients to check the facts about their emotions. This ability makes patients realize whether their emotional reactions fit the facts of the situation and increases control over the access to negative cognitions activated by negative mood states.

The current emotion regulation skills intervention taught patients how to solve problems, define alternatives, and choose appropriate solutions. Thus, the patient can select a prompting event that triggered a painful emotion or an event that was changed and turn the event into a problem to be solved [39, 66]. Developing problem-solving skills can be used for the reappraisal of the situation, finding practical and creative solutions, and helping patients to be more independent and develop initiative for planning their lives [67]. The intervention enhanced patients' ability to refocus on planning and positive reappraisal.

Higher scores of emotion awareness after the emotion regulation intervention may be explained by the change in emotion recognition achieved after training. It was found that patients with depression who can describe, observe, and name their emotions help themselves to regulate emotions [68]. The advantage of emotional information and integrated cognitive processes is that they allow conscious awareness of emotions. Awareness of emotion helps the patient's emotional state and, therefore, offers the flexibility of emotional response to help achieve adaptational success.

After the emotion regulation skill training, the current study revealed a diminution of "non-acceptance" among the study subjects. This can be explained by developing mindfulness skills, which enhance patients’ ability to focus on their current emotions and accept their state [69]. Mindfulness practices emphasize a non-judgmental attitude toward the meaning of a patient's experiences, learning not to label their thoughts, feelings, or experiences as good or bad and trying not to change or resist them in any way [70, 71]. Mindfulness skills help patients cope with depression by redirecting them from negative thoughts and accepting difficult emotions. Patients can regulate their emotions regarding their thoughts and feelings about what is present. Along the same line, being mindful of emotions helps to switch to more positive mindsets and work towards being emotionally regulated.

Additionally, present-focused awareness is a core feature of mindfulness, which is thought to transform how momentary experiences are observed and processed. It facilitates engagement with emotional stimuli and thus enriches the experience while reducing emotional reactivity [69]. Consequently, mindfulness skills have a better effect, resulting in acceptance and awareness of emotion. In the present study, patients scored lower in these difficulties after emotion regulation skills intervention.

Furthermore, mindfulness skills could be contributing factors in handling patients’ self-blame strategy after conducting emotion regulation skills. Evidence for the beneficial effects of mindfulness on lowering self-blame through activation of regions typically involved in negative self-judgments and self-blame [70–73]. Mindfulness skills teach patients to focus on the current moment without negatively criticizing themselves. In this context, mindfulness training enables patients with depression to develop their capacity to respond to negative self-judgments with a friendly, open, and self-compassionate stance.

A significant difference was found between subjects’ scores in impulse control after emotion regulation intervention. This result could also be attributed to the development of mindfulness skills. Concentrating on the current moment reduces emotional impulses and anxiety. A study by Castelli and Tesio supports this explanation. They found that mindfulness helps patients view anger as just one of many other emotional experiences, decreasing the impulse of anger. Mindful individuals are more willing to control negative internal emotions [31, 73, 74].

The current study's subjects showed a positive change in goal-directed behavior after emotion regulation skills intervention. This could be attributed to teaching patients the skill of accumulation of positive emotions. This skill enhances patient motivation by reaching goal-directed behaviors and enhancing positive expectations toward their day. Patients were asked to list positive events they could do in their day to improve their psychological status. Hopefully, these events motivate patients to think, which can change their mindset and behavior through goal-directed actions.

### Limitation

Despite the strengths of the study’s positive efficacy of emotion regulation skills on depression, there is a need to mention such limitations. The limitations of this study are primarily related to its quasi-experimental design and sample size. The non-randomized design introduces potential biases, limiting the ability to establish a causal relationship between the intervention and the observed outcomes. This design choice inherently includes the risk of selection bias, where differences between the intervention and control groups may influence the results beyond the intervention itself. Additionally, the relatively small sample size limits the generalizability of the findings to a broader population, reducing the conclusions' robustness. Also, we did not measure symptom severity, future research should aim to assess the severity of depression symptoms in conjunction with emotion regulation interventions. Given the fluctuating nature of MDD and its impact on treatment outcomes, incorporating a detailed evaluation of depression severity can provide deeper insights into the efficacy of emotion regulation strategies.

## Implication

The findings of this study have several important implications for patient care in the management and treatment of depression. Healthcare providers should routinely assess emotion regulation difficulties in patients with depression to identify specific areas of struggle. They are integrating personalized treatment plans that target individual emotion regulation difficulties. A holistic approach, combining emotion regulation skills training with traditional pharmacological and psychotherapeutic interventions, addresses both emotional and cognitive aspects of depression, leading to better outcomes. Additionally, support groups or workshops focused on emotion regulation can offer patients a sense of community, a platform to share experiences, and opportunities to practice new skills. Finally, training healthcare providers in emotion regulation techniques and their application in clinical practice is essential to enhance their ability to support patients effectively.

## Conclusion

These results highlight that individuals with depression face considerable challenges in emotion regulation, characterized by more significant difficulties and a higher reliance on maladaptive strategies. Emotion regulation skills training significantly alleviates these difficulties, promoting adaptive strategies among patients. The findings suggest that the emotion regulation intervention has the potential to improve emotion regulation difficulties and cognitive emotion regulation strategies among patients with depression. These findings highlight the importance of incorporating emotion regulation interventions into the treatment of depression to enhance therapeutic outcomes.

## Data Availability

The datasets used and analyzed during the current study are available from the corresponding author upon reasonable request.
